# Cinema, censorship and educational impact: a semiotic and empirical analysis of the compilation–oral history documentary “Yollara Düştük” (We Hit the Road)

**DOI:** 10.3389/fpsyg.2026.1872700

**Published:** 2026-07-17

**Authors:** Onur Ülkü, Fevzi Kasap

**Affiliations:** 1 Department of Media and Communication Studies, Near East University, Faculty of Communication, Lefkoşa/Mersin, Türkiye; 2 Department of Radio-Television-Cinema, Near East University, Faculty of Communication, Lefkoşa/Mersin, Türkiye

**Keywords:** documentary cinema, censorship, semiotic analysis, educational impact, scale development, mixed methods, hegemony, Turkish

## Abstract

**Introduction:**

Compilation-oral history documentaries operate at the intersection of ideological struggle and educational praxis, yet their dual function as counter-hegemonic texts and pedagogical tools remains under-theorized. This two-phase study examines the 2014 Turkish documentary Yollara Düştük through semiotic and educational lenses to investigate how hegemonic control is contested in compilation documentaries and how such films influence university audiences' perceptions of censorship, motivation, and beliefs about success.

**Methods:**

Phase 1 employed a critical-semiotic analysis drawing on Peirce's sign model, Eco's concept of the “open work,” Barthes' myth theory, and Gramsci's theory of hegemony to examine how archival images, editing strategies, and oral testimonies transform symbolically within the documentary text. Phase 2 utilized an explanatory sequential mixed-methods design. A newly developed and validated 24-item instrument-the Documentary Cinema Educational Impact Scale (BSES)-was administered to 440 university students and faculty members across three dimensions: Perception of Censorship and Attitude Against It, Educational Motivation, and Beliefs in Success in Life. Model fit was assessed using confirmatory factor analysis. Qualitative interviews (*n* = 30) were subsequently conducted, and joint display analysis was used to integrate quantitative and qualitative findings.

**Results:**

Semiotic analysis revealed that censorship functions as a semiotic struggle in which archival traces are recoded within counter-hegemonic narratives. The opening sequence (00:00–03:15) employs static shots of closed cinema doors and silence to symbolize suppression, while the archival montage (12:40–18:10) forges temporal links between 1977 and 2014 footage, transforming historical traces into symbols of resistance. Quantitatively, the BSES demonstrated excellent model fit (χ^2^/df = 1.95, RMSEA = 0.052, CFI = 0.95, TLI = 0.94, SRMR = 0.045). Educational motivation emerged as the strongest outcome ($\bar{x}$ = 3.95), followed by beliefs in success in life ($\bar{x}$ = 4.08), with structural censorship perception showing more moderate effects ($\bar{x}$ = 3.72). Qualitative findings indicated that the film reinterprets repressed events by recontextualizing archival footage and constructing a counter-hegemonic discursive space. Joint display analysis confirmed that the documentary enhances individual motivation but exerts limited influence on perceptions of structural censorship.

**Discussion:**

Compilation-oral history documentaries function simultaneously as sites of ideological struggle and as educational tools that enhance motivation and success beliefs among university audiences. However, their impact appears largely confined to symbolic awareness and individual transformation rather than catalyzing structural or institutional change. These findings suggest that while such documentaries effectively mobilize counter-hegemonic meaning-making at the semiotic level, their educational efficacy in fostering critical consciousness about systemic censorship remains bounded. Future research should explore pedagogical strategies that bridge the gap between symbolic awareness and structural critique.

## 1 Introduction

Cinema entered our lives with the cinematograph invented by the Lumière Brothers in 1895, creating a new type of sign: the moving image (Kiraç, 2018). According to Peirce (1931–1958) classification, the cinematographic image operates at the iconic (visual similarity), indexical (physical link to reality), and symbolic (meaning through cultural codes) levels. At the indexical level, the image functions as a “trace” or “footprint” of a real historical event, establishing a physical, causal connection between the sign and its object (e.g., the actual light rays reflected from the 1977 march participants onto the film stock). At the iconic level, the image resembles its object through visual similarity (e.g., the recognizable faces of the marching cinema workers). At the symbolic level, cultural and ideological conventions imbue the image with meanings that exceed its immediate visual content (e.g., the march as a symbol of democratic resistance). This progression—from indexical trace to iconic recognition to symbolic interpretation—establishes the conceptual continuity between early photographic realism and the later emergence of compilation documentary practices, wherein archival footage is extracted from its original indexical context and subjected to symbolic re-encoding through montage and narrative framing. Cinema functions not only as a means of entertainment but also as a semiotic space where social systems of meaning are produced, circulated, and negotiated.

Documentary cinema, in particular, is a “clear text” (Eco, 1976) in semiotic terms as “the creative treatment of actuality” (Grierson, 1933). Grierson's original formulation emphasizes the transformative process through which recorded reality is shaped into a meaningful documentary text through selection, organization, and representation. Barthes (1977) shows that images function at two levels: the denotative (conveying literal or descriptive information) and the connotative (loaded with cultural, ideological, and symbolic codes). Historical images, in Peirce's words, first appear as “indexical traces”; however, within the context of montage and narrative, they enter secondary semiosis—a process whereby the original documentary “trace” is lifted from its initial context and re-embedded within a new symbolic system—and become ideological myths (Barthes, 1972). In this context, censorship should be understood not only as institutional prohibition but also as the semiotic regulation of meaning production, that is, the limitation of the “tellable” and “visible” spaces (Foucault, 1972).

Documentary cinema becomes a field of semiotic struggle in which hegemonic meanings are either reinforced or distorted. However, it is essential to acknowledge that documentary cinema is not inherently emancipatory. As the history of propaganda cinema demonstrates, documentaries may equally serve to naturalize and reinforce hegemonic ideologies. Leni Riefenstahl's *Triumph of the Will* (1935) transformed the indexical traces of the Nuremberg rallies into mythic symbols of Aryan supremacy, while Soviet montage films under Stalin recoded archival footage to legitimize state authority (Nichols and Baron, 2024). These examples demonstrate that the same semiotic mechanisms of recoding can serve both hegemonic and counter-hegemonic ends. Gramsci's (1971) concept of hegemony suggests that the ruling classes establish power over society not only by force, but by producing consent through cultural and ideological leadership. Documentary cinema intervenes in the semiotic production of this consent and questions the ideological structures naturalized as “common sense.” Recent studies in visual semiotics and critical media studies emphasize that documentary images are not neutral recordings but rather spaces for active meaning-making (Hjelm, 2021; Kruk, 2025; Pellitteri, 2024).

The 2014 documentary *Yollara Düştük* (We Hit the Road) is an important example that questions the relationship between cinema and censorship in Turkey by retelling the 1977 Istanbul-Ankara march through archival footage and current testimonies (Yeşil, 2014). The documentary combines archival footage of the 1977 Istanbul-Ankara march with contemporary testimonies to perform a secondary semiosis of the historical event. The 1977 march, in which approximately 400 cinema workers walked from Istanbul to Ankara to protest censorship and demand freedom of expression, represents a pivotal moment in Turkish cinema history that has been largely erased from official narratives.

The documentary itself became a victim of the very censorship it documented. When the documentary *Bakur* was prevented from screening at the Istanbul Film Festival in 2015, director Deniz Yeşil completed *Yollara Düştük* in solidarity and uploaded it to digital platforms, bypassing the bureaucratic “Operational Certificate” requirement imposed by the Ministry of Culture and Tourism. This ironic twist—a documentary about censorship being itself subjected to censorship—exemplifies the ongoing semiotic struggle over meaning in Turkish cinema.

Despite its critical importance, the educational impact of such documentaries on university audiences remains underexplored. Recent studies show that documentary films may increase student motivation and contribute positively to academic success in educational environments when the content aligns with students' critical interests and the screening is accompanied by structured discussion (Özkan and Arikan, 2009; Küçükosmanoglu, 2015). It has been determined that education-themed films, in particular, increase teacher candidates' intrinsic motivation and positively change their attitudes toward the profession (Küçükosmanoglu, 2015). However, these studies usually focus on a single dimension (motivation or attitude); scales that holistically examine the multidimensional impact of documentary cinema (perception of censorship, motivation, and beliefs in success) are limited.

In Northern Cyprus, the multicultural nature of university students and their diverse experiences with censorship further complicate these dynamics. Previous studies have shown that university students' attitudes toward censorship in Turkey are contradictory and inconsistent; despite high approval rates, they experience ambivalence at both the practical and ideological levels (Özkan and Arikan, 2009). This situation reveals that the educational impact of documentary cinema should be examined contextually.

### 1.1 Purpose of the study and research questions

This study aims to examine the mechanisms of meaning production and counter-hegemonic semiosis in documentary cinema under censorship from a semiotic perspective, while simultaneously investigating the documentary's educational impact on university students and faculty members. The study is structured in two phases:

*Phase 1 (Semiotic-Theoretical):* How does compilation documentary cinema, under censorship conditions, construct counter-hegemonic systems of meaning through the semiotic transformation of archival footage, and how do these meanings undergo semiosis again on digital platforms?

*Phase 2 (Empirical-Educational):*

- Do the perceptions of censorship, educational motivation, and beliefs about success in life after watching the film differ according to gender, education level, and status (student/instructor) variables?
- What are the variables that predict these three dimensions?
- How do participants experience and interpret the film?
- How does the film transform participants' perception of censorship and their views on life?
- What is the relationship between quantitative scores and qualitative themes?

### 1.2 The documentary: Yollara Düştük (We Hit the Road)

*Yollara Düştük* (Director: Yeşil, 2014) exhibits a semiotically multi-layered structure. Rather than a pure compilation documentary, Yollara Düştük is more accurately described as a hybrid compilation–oral history documentary. While archival footage provides the historical and indexical foundation of the narrative, contemporary testimonies by participants and cultural figures function as interpretive mechanisms that guide audience understanding of the archival record. The documentary combines archival footage of the 1977 Istanbul-Ankara march with contemporary testimonies to perform a secondary semiosis of the historical event. The semiotic structure can be analyzed as follows:

(a) *Indexical level:* images of the 1977 march establish a physical connection as a “trace of reality.” The images were taken at a specific time (1977) and place (the Istanbul-Ankara Road), which establishes a physical, causal link between the signifier and the signified, in accordance with Peirce's definition of the “index.”
(b) *Iconic level:* images present the visual resemblance of the march: cinema workers, roads, banners, crowds. This iconic level initiates the viewer's process of recognition and identification.
(c) *Symbolic level:* however, montage and narrative context imbue these images with new symbolic meanings. The 1977 March, in its 2014 production, is recoded as “a symbol of resistance to censorship.” Figures such as Tarik Akan, Fatma Girik, and Cüneyt Arkin, as cinema workers of Yeşilçam's “golden age,” become symbols of the “collective memory” of Turkish cinema history.

The following semiotic transformation is demonstrated through concrete audiovisual evidence: in Sequence B (12:40–18:10), archival footage of the 1977 march is juxtaposed with contemporary interviews. The indexical trace of the original march footage—its physical connection to the historical event—is preserved through the film stock's materiality (grain, color degradation, period-specific clothing). However, through syntagmatic association with 2014 testimonies, these indexical traces undergo symbolic recoding: the march is no longer merely a historical event but becomes a symbol of ongoing democratic resistance. The editing rhythm accelerates during crowd sequences and decelerates during individual testimony shots, creating a dialectical movement between collective action and personal memory that constructs the film's counter-hegemonic narrative.

Director Deniz Yeşil's discourse explains this semiotic transformation:

“*In 1977, all social opposition was on the streets… The streets were not used to seeing filmmakers. Still, the directors had many problems… Four hundred cinema employees organized a three-day march from Istanbul to Ankara… The cinema of this country is not apolitical; it is not indifferent to problems.”*

This discourse semiotically shows the practice of “oppositional coding”: a “political” and “oppositional” alternative meaning is produced against the “apolitical” and “entertainment-oriented” hegemonic image of Turkish cinema.

The documentary functions as a semiotic “censorship text”: while it addresses censorship, it also becomes a victim of it. This situation can be examined at three semiotic levels:

(a) *Historical censorship (semiotic archive):* the documentary archives the semiotic traces (documents, photographs, news) of censorship practices in the 1960s−70s. These traces are semiotic evidence of the original “regime of discourse” (Foucault, 1972).
(b) *Festival censorship (prevention of semiotic circulation):* what happened during the 2014–2015 festival period shows the prevention of the circulation of meaning in “institutional spaces” (festivals). At the Antalya Golden Orange Film Festival, Reyhan Tuvi's documentary “Until the Face of the World Becomes Love” was deemed “semiotically risky,” leading to the cancellation of 12 of 15 films. The screening of the documentary *Bakur* at the Istanbul Film Festival triggered the production of *Yollara Düştük* for solidarity.
(c) *Bureaucratic censorship (semiotic coding):* the “Operational Certificate” requirement aims at the “pre-coding” of meaning production. The Ministry of Culture and Tourism's evaluation with vague criteria such as “national interests” and “public morality” aims to reproduce the hegemonic “common sense semiotically.”

Director Yeşil's opposition to this requirement is a semiotic “coding struggle”:

“*I do not have a certificate, even if I did, I would not submit it… This is direct censorship, blocking the film.”*

Türkan Soray's words in the documentary show the semiotic intensification of this resistance:

“*How long will this pressure and censorship continue? It had to end and explode somewhere.”*

The metaphor of “explosion” expresses the “explosion into semiosis” of semiologically repressed meanings. Yeşil's decision to upload the film to digital platforms expands the semiotic field of this “explosion”:

“*We also had to explode, and that explosion is happening wonderfully.”*

## 2 Theoretical framework

### 2.1 Documentary cinema: semiotic definition, historical development, and social function

From a semiotic point of view, documentary cinema deals with the multi-layered sign system of reality. Within the framework of Peirce (1931–1958) tripartite model: (a) at the iconic level, the image bears a resemblance to reality; (b) at the indexical level there is a physical connection (trace of reality) between the moment of attraction and the event shown; (c) at the symbolic level, historical and ideological meaning is attributed through cultural codes. In compilation documentaries, the tension between these three levels is particularly evident: archival footage is extracted from its original indexed context and subjected to symbolic re-encoding within the framework of the new narrative.

Oskay (2001) defines documentary film as “an art form that resists oblivion, cruelty, and unhappiness caused by a particular form of human relationship with nature and other people.” This definition emphasizes that documentary is not only the production of signs, but also the re-semiosis of “forgotten” meanings, that is, the practice of semiotic resurrection. In Grierson's (1933) classic definition, documentary cinema is “the creative treatment of actuality”. This “treatment,” in Eco's (1976) words, points to the “open” structure of the text: documentary is not a closed, certain meaning, but a semiotic structure that invites multiple readings.

The origins of documentary cinema date back to the silent era. The subtitles of silent cinema are early semiotic interventions that add meaning to the image through written text. In 1927, with the film “The Jazz Singer,” cinema entered the sound era; this transition means the semiotic inclusion of a new layer (auditory signs) in the production of meaning (Abisel, 2010).

During World War II, when documentary cinema was used as a propaganda tool, semiotic manipulation was at its height. In Nazi Germany and Fascist Italy, ideological recoding was carried out at the symbolic level, preserving the iconic and indexical levels of reality. For instance, Leni Riefenstahl's *Triumph of the Will* (1935) transformed the indexical traces of the Nuremberg rallies into mythic symbols of Aryan supremacy, while Soviet montage films under Stalin recoded archival footage to legitimize state authority (Nichols and Baron, 2024). These examples demonstrate that the same semiotic mechanisms of recoding can serve both hegemonic and counter-hegemonic ends. The 1960s is the period when the “creative interpretation” potential of documentary cinema was semiotically recognized. The direct cinema (observational documentary emphasizing non-intervention and synchronous sound, as in the works of the Maysles brothers) and cinéma vérité (participatory documentary acknowledging the filmmaker's presence and provoking reality, as in Jean Rouch's *Chronicle of a Summer*) movements reveal that the director's observer position is itself a practice of producing signs. In documenting social movements, young filmmakers in Turkey discover that images are not just indexical evidence but symbolic calls to action (Çelikcan, 2020).

Today, digital technologies are transforming the semiotic structure of documentary cinema. Poell et al. (2024) show that cultural production in the platform age operates within new semiosic orders. Pellitteri (2024) emphasizes how digital documentaries create new communities of meaning through algorithmic visibility and participatory interpretation practices. Kruk (2025) applies Peirce's classification of signs to documentary analysis, illustrating how montage practice accelerates transitions among iconic, indexical, and symbolic levels.

### 2.2 Compilation documentary: semiotic definition, historical origin, and social function

“Compilation documentary” is one of the most semiotically complex documentary genres. While other genres—such as observational (direct cinema), participatory (cinéma vérité), expository (authoritative voice-of-God narration), and reflexive (self-aware examination of the documentary process)—each present distinct semiotic challenges, the compilation documentary is unique in its radical dependence on pre-existing sign systems. This genre is based on the recontextualization of previously produced sign systems (archival images), i.e., secondary semiosis. In Peirce (1931–1958) terms, the archival image first functions as a “trace of reality” (index); but when it is incorporated into the new narrative context, this indexical link weakens, and the image takes on new symbolic meanings.

However, *Yollara Düştük* does not fully conform to the conventional characteristics of a purely documentary film. According to the definitions established in documentary studies, the film exemplifies a hybrid genre: it integrates elements of the compilation documentary through archival footage and montage, while foregrounding the features of the oral history documentary, with its personal testimonies and retrospective accounts. Explicitly defining *Yollara Düştük* as a hybrid compilation–oral history documentary clarifies its placement within established genre frameworks and enables a more precise analysis of its narrative and formal strategies.

Dziga Vertov's “Man with a Film Camera” (1929) is an early example that laid the semiotic foundations of the compilation documentary. Through montage, Vertov breaks down the original indexed contexts of images and produces new dialectical meanings. This is the practice of “slippage of meaning” semiologically: the same image carries different symbolic loads in different contexts.

Compilation documentaries are inherently “intertextual” structures (Kristeva, 1969; Tsang, 2013). Archival images join the new narrative through the semiotic “memory” they carry from their prior use. Eco's (1976) concept of “open work” is critical here: the compilation documentary reopens pre-existing texts (archival footage), inviting multiple readings and interpretations.

In Turkey, the compilation documentary genre has gained prominence in the semiotic reconstruction of political memory. *Yollara Düştük* combines archival footage of the 1977 protest with contemporary testimonies, establishing a semiotic “intertemporal” link between the past and the present. Sesame (2015) emphasizes the function of such documentaries as “semiotic storage and re-enactment of collective memory.”

Compilation documentaries can serve as a form of semiotic resistance to censorship and hegemony. Censorship protects hegemonic “common sense” by preventing certain meaning-making practices. However, the compilation documentary, through the recontextualization of archival footage, restarts the semiosis of repressed meanings. Tsang (2013) defines this process as “the semiotic performance of political memory.” Kruk (2025) shows how the montage practice of compilation documentaries semiotically reorganizes the processes of “production, circulation, and consumption of meaning.”

In *Yollara Düştük*, the rearrangement of the 1977 protest footage involves the following transformations semiologically speaking: (a) while preserving the indexical context of the original footage (the political circumstances of 1977); (b) within the framework of the new narrative (2014 production) the layers of symbolic meaning are recoded; (c) By interpreting this dual-layered system of signs, the viewer makes a semiotic “transition” between historical and contemporary political contexts.

### 2.3 Censorship and hegemony: semiotic conceptual framework and social implications

Censorship should be understood semiotically as a “regime of discourse” (Foucault, 1972): one modality among several that regulates what can be said, seen, and remembered. As Foucault emphasizes, liberal bourgeois democracies also operate through regimes of discourse—albeit more diffuse ones—in which power circulates through institutional practices rather than through explicit prohibition alone. This regime naturalizes hegemonic “common sense” by drawing the limits of meaning production (Gramsci, 1971; Barthes, 1972).

The semiotic definition of censorship operates on two levels: (a) the “narrow” definition is defined as government agencies controlling political or moral content; (b) the “broad” definition is the indirect regulation of meaning production practices by hegemonic structures, namely semiosis (Jansson, 2022). This study adopts the broad definition and treats censorship as a semiotic struggle over meaning. The broad definition can be identified through critical discourse analysis, ideological critique, and the examination of structural silences—what is systematically absent from media and educational narratives—rather than through explicit prohibitions alone. For instance, the consistent omission of labor history from mainstream curricula or the algorithmic demotion of dissident content constitutes broad censorship even in the absence of a specific ban order.

Gramsci's (1971) concept of hegemony explains the semiotic functioning of censorship. Hegemony is the establishment of power by the ruling classes over society by producing “consent”; this consent is achieved through cultural and ideological means, through the internalization of systems of meaning. Censorship is the mechanism that ensures that these systems of meaning do not open up to alternative semiosis (counter-narratives).

Barthes's (1972) concept of “myth” explains the semiotic naturalization of hegemonic meaning. Myth is the semiotic system that makes historical and ideological meaning seem “natural.” By disrupting these myths, documentary cinema questions the “naturalness” of images and makes historical production processes visible.

Hall's (1980) coding/decoding model explains the semiotic dynamics of censorship and resistance. Hegemonic coding presents dominant meanings as “preferred reading”; however, the audience may have an “oppositional reading” or “negotiated reading.” Censorship tries to limit these reading positions, but the compilation documentary expands the possibilities of countercoding through the recontextualization of archival footage.

#### 2.3.1 The semiotic relationship between censorship and hegemony

Censorship and hegemony are semiotically linked as practices of “regulation of meaning.” Hegemony aims to prevent the social prevalence of certain systems of meaning; censorship aims to prevent the semiosis of alternative systems of meaning. Zaimoglu (2016), while examining the role of the media in the construction of hegemony, shows that censorship operates not only directly but also semiotically through “information manipulation” and “distorted news.”

While analyzing the role of the military and the media in the construction of hegemony, Korkmaz (2016) uses the expanded definition of censorship as “interventions in the production, circulation, and consumption processes of meaning.” With this perspective, censorship regulates not only the question of “what to say” but also “how to say it,” “by whom,” and “in what context.”

#### 2.3.2 Semiotic management of media, censorship, and the circulation of meaning

Digital media has transformed the circulation of meaning and the fields of semiotic struggle. Poell et al. (2024) examine how “semiotic regimes of cultural production” have changed in the age of the platform. Platforms function as bidirectional semiotic agents, simultaneously (a) accelerating the circulation of hegemonic meanings and (b) making it possible for counter-hegemonic meanings to gain visibility.

Oser (2022), while examining how the use of digital media affects political participation, proposes the concept of “semiotic citizenship”: participation in the production of meaning on digital platforms is the semiotic construction of political subjectivity.

However, digital platforms do not fully deliver on the promise of “semiotic democratization.” Cardullo and Kitchin (2025) show how platform governance regulates the production of meaning through “visibility policies.” Algorithmic filtering, as a new form of “soft censorship,” encourages the circulation of certain meanings while making others invisible. However, we acknowledge that algorithmic filtering differs from traditional censorship in important respects: unlike state censorship, algorithms do not explicitly prohibit content but rather hierarchize visibility through engagement metrics, commercial imperatives, and opaque ranking systems. In this sense, algorithmic filtering resembles the editorial logic of legacy media (television, radio, and newspapers), which always already selected and prioritized certain meanings over others. What distinguishes algorithmic “soft censorship” is its automated, non-transparent, and individually customized nature, which renders the gatekeeping mechanism less visible and therefore more difficult to contest than the explicit editorial decisions of broadcast-era media (Cardullo and Kitchin, 2025).

#### 2.3.3 Education and cultural hegemony: semiotic dimension

The semiotic effects of censorship also spread to education and cultural production. In the Turkish context, the education system functions as a mechanism for transmitting hegemonic systems of meaning. Official narratives in history classes are semiotically coded as “natural” and “inevitable,” while alternative narratives—such as the 1977 cinema workers' march—are systematically excluded from curricula, constituting a form of structural or broad censorship. In their critical media literacy studies, Dagtaş and Okuroglu (2018) emphasize how cultural hegemony is “semiotically reinforced” through media content. In popular-culture products, the emphasis on certain values and the marginalization of alternative narratives sustain hegemonic control.

Censorship practices against documentary cinema in Turkey embody these semiotic dynamics. The refusal to award at the Antalya Golden Orange Film Festival (2007) on the grounds of “not finding works that meet the criteria for professional documentaries” is a rhetorical strategy that ensures the “semiotic legitimacy” of censorship. Ulutak (2007) defines documentary cinema as “a platform where different voices are expressed semiologically, sensitive issues are negotiated, and taboos are broken.” Işikman (2015) emphasizes the “capacity of documentary cinema to question social memory and produce alternative narratives semiotically.”

### 2.4 Documentary cinema and educational impact

Documentary cinema operates at iconic (visual similarity), indexical (physical connection to reality), and symbolic (meaning through cultural codes) levels within the framework of Peirce (1931–1958) triadic sign model. Eco's (1976) concept of “open work” emphasizes that documentary is a semantic structure that invites multiple readings by the audience. This feature makes documentary cinema a powerful tool in educational settings (Wildwood Media, 2025).

According to Grierson's (1933) classical definition, documentary cinema is “the creative treatment of actuality.” This treatment can create both emotional engagement (empathy, anger, sadness) and cognitive transformation (knowledge acquisition, perspective shift) in the audience (Barchas-Lichtenstein et al., 2020). Documentaries, especially those on social issues, can prompt viewers to take behavioral actions, such as seeking information, donating, and engaging in community action (Participant Media, 2014). For example, 89% of those who watched the documentary “Girl Rising” said they learned more about the social issues in the film; 75% experienced a high level of emotional engagement; and 50% took community-driven action after watching it (Participant Media, 2014). These statistics align with Bandura's (1977) self-efficacy theory and Küçükosmanoglu (2015) findings on educational motivation, suggesting that documentary cinema operates through emotional-cognitive pathways that enhance viewers' sense of agency and critical awareness.

### 2.5 Motivation and success beliefs

Self-efficacy theory (Bandura, 1977) suggests that individuals' beliefs about their capacity to accomplish certain tasks drive behavior. Documentary films can increase the audience's self-efficacy by highlighting success stories and struggles. In particular, films such as *Yollara Düştük* can strengthen the audience's belief that “I can succeed too” through the determination and solidarity of the cinema workers who participated in the 1977 march. Nevertheless, we recognize that this effect is historically and contextually contingent. As critical media scholars caution, documentaries memorializing past labor struggles may simultaneously inspire and demoralize viewers: they may draw strength from historical solidarity while concluding that such collective action is no longer viable under contemporary neoliberal conditions (Barchas-Lichtenstein et al., 2020). Our qualitative data partially reflect this ambivalence—participants reported heightened personal motivation yet expressed skepticism about structural change—suggesting that vicarious self-efficacy operates primarily at the individual psychological level rather than at the level of political mobilization.

It has been shown that education-themed films increase teacher candidates' intrinsic motivation and positively change their attitudes toward the profession (Küçükosmanoglu, 2015). In this study, the intrinsic motivation scores of the experimental group ($\bar{X}$= 38.81), who watched education-themed films, were significantly higher than those of the control group ($\bar{X}$ = 36.67; *t* = −3.832, *p* < 0.05) (Küçükosmanoglu, 2015). In addition, the “love” sub-dimension scores of the pre-service teachers who watched movies ($\bar{X}$ = 99.89) increased significantly compared to the control group ($\bar{X}$ = 89.17; *t* = 3.330, *p* < 0.05) (Küçükosmanoglu, 2015).

### 2.6 Hypotheses

H1: The recontextualization of archival footage in the compilation documentary enables the semiotic “disassembly” of hegemonic myths, producing new layers of meaning at the level of Peirce's “symbol.” This hypothesis assumes a transformation in the process of meaning production, involving the removal of images from their original indexical contexts and their symbolic recoding within the framework of the counter-hegemonic narrative.

H2: The circulation of censored documentary film on digital platforms represents a semiotic expansion from the limited production of meaning (the closed mechanisms of festivals) to boundless communities of interpretation, in line with Eco's concept of “open work.” This hypothesis supposes the production and consumption of digital circulation within new semiospheres, that is, semiotic democratization.

H3: The documentary director's practice functions as a semiotic “intervention” against hegemonic discourse within Hall's “counter-coding” model. This hypothesis assumes that the director's practices of selecting, editing, and recontextualizing archival footage constitute a conscious intervention in the ideological dimension of meaning production.

H4: The documentary *Yollara Düştük* significantly increases educational motivation and beliefs in personal success among university students and faculty members.

H5: The perception of censorship and attitude against it differ significantly according to demographic variables (gender, status, age, department).

## 3 Methodology

This study employs a mixed-methods design that combines semiotic analysis and qualitative content analysis for Phase 1, and an explanatory sequential mixed-methods design for Phase 2. The methodology focuses on the study of meaning-production processes, namely, semiosis, alongside the empirical measurement of educational impact.

### 3.1 Phase 1: semiotic analysis methodology

#### 3.1.1 Primary data collection: interviews with the director

The interviews were conducted in a semi-structured format to examine “discourse production practices” from a semiotic perspective. Director Deniz Yeşil's statements were analyzed in the context of (a) semiotic preferences in the selection of archival footage, (b) meaning production strategies in montage practice, and (c) the semiotic meaning of digital circulation in the face of censorship.

However, consistent with the reviewer's recommendation to treat the film as a film, the primary analytical evidence in Phase 1 derives from close analysis of the documentary's audiovisual text rather than from director interviews. Interview data are used only as supplementary contextual material, not as primary evidence for semiotic claims.

Interview analysis aims to examine “discursive construction.” Dahal (2024) examines the semiotic interplay between language, ideology, and power, showing that discourse functions as an “active space for meaning production.” Parsa (2012) proposes examining the director's discourse as a “coding practice” through structural analysis methods in cinema semiotics.

#### 3.1.2 Film content analysis: semiotic framework

The analysis of the film requires multi-layered semiotic examination:

(a) *Visual level:* iconic, indexical, and symbolic functions of archival images; the contribution of color, composition, and movement elements to the production of meaning.
(b) *Auditory level:* the discursive structure of the testimony discourse; the emotional-semantic impact of music and sound design.
(c) *Assembly level:* new semantic relationships produced by combining images (syntagmatic association) and juxtaposing (paradigmatic selection).
(d) *Narrative level:* renarrativization of the historical event (1977 March) and construction of the counter-hegemonic narrative structure.

This analysis follows the semiotic framework developed by Tsang (2013) and Kruk (2025) for compilation documentaries. Kruk (2025) applies Peirce's 10-gauge class system to documentary analysis, showing how montage accelerates semiotic transformation. Pellitteri (2024) examines the semiotic functioning of digital documentaries within the “platform logic.”

#### 3.1.3 Semiotic examination of digital platforms

Digital platforms are conceptualized as “socio-technical systems” in which the processes of production, circulation, and consumption of meaning are reorganized (Poell et al., 2024). This analysis examines how (a) platform algorithms regulate the visibility of meaning, (b) how user comments and shares contribute to the reproduction of meaning, and (c) how digital circulation reinforces the “open text” structure.

Cardullo and Kitchin (2025) illustrate how platform governance shapes the practices of “semiotic citizenship.” Oser (2022) emphasizes the participatory semiosis dimension of digital media use.

#### 3.1.4 Close audiovisual semiotic analysis and hypothesis evaluation procedure

To address the relationship between cinematic form and meaning production, the study incorporated a close audiovisual semiotic analysis of three representative sequences selected through purposive sampling. Selection criteria included (a) centrality to the documentary's narrative structure, (b) intensive use of archival footage and montage, and (c) explicit engagement with censorship and collective memory.

The documentary (runtime: approximately 52 min) was viewed repeatedly and divided into analytical units. Three key sequences were selected:

*Sequence A (Opening Sequence):* closed cinema doors, empty exhibition spaces, and introductory voice-over narration.

*Sequence B (Historical March Sequence):* archival footage documenting the 1977 Istanbul–Ankara march and its integration with contemporary testimonies.

*Sequence C (Censorship and Festival Sequence):* interviews, archival documents, and references to contemporary censorship practices surrounding documentary circulation.

The analytical framework consisted of four coding dimensions:

1. *Visual Composition*

- Shot scale
- Camera movement
- Framing
- Archival image quality
- Spatial organization

2. *Montage Structure*

- Temporal juxtaposition
- Archival/contemporary transitions
- Editing rhythm
- Repetition patterns

3. *Sound Design*

- Voice-over narration
- Testimony discourse
- Music
- Silence
- Sound-image interaction

4. *Semiotic Function*

- Indexical meaning
- Iconic meaning
- Symbolic meaning
- Myth construction/deconstruction

Hypothesis evaluation followed transparent analytical criteria.

H1 was considered supported when archival images were demonstrably removed from their original historical context and assigned new symbolic meanings through montage and narrative framing.

H2 was considered supported when evidence demonstrated the expansion of meaning circulation beyond traditional exhibition spaces through digital distribution and audience participation.

H3 was considered supported when editing decisions, archival selection, and circulation strategies could be identified as deliberate counter-hegemonic interventions within Hall's encoding/decoding framework.

Rather than treating hypotheses as statistically confirmed, the study interprets them as analytically supported through triangulation of audiovisual evidence, textual analysis, and production context.

### 3.2 Phase 2: explanatory sequential mixed methods design

**Figure 1:**
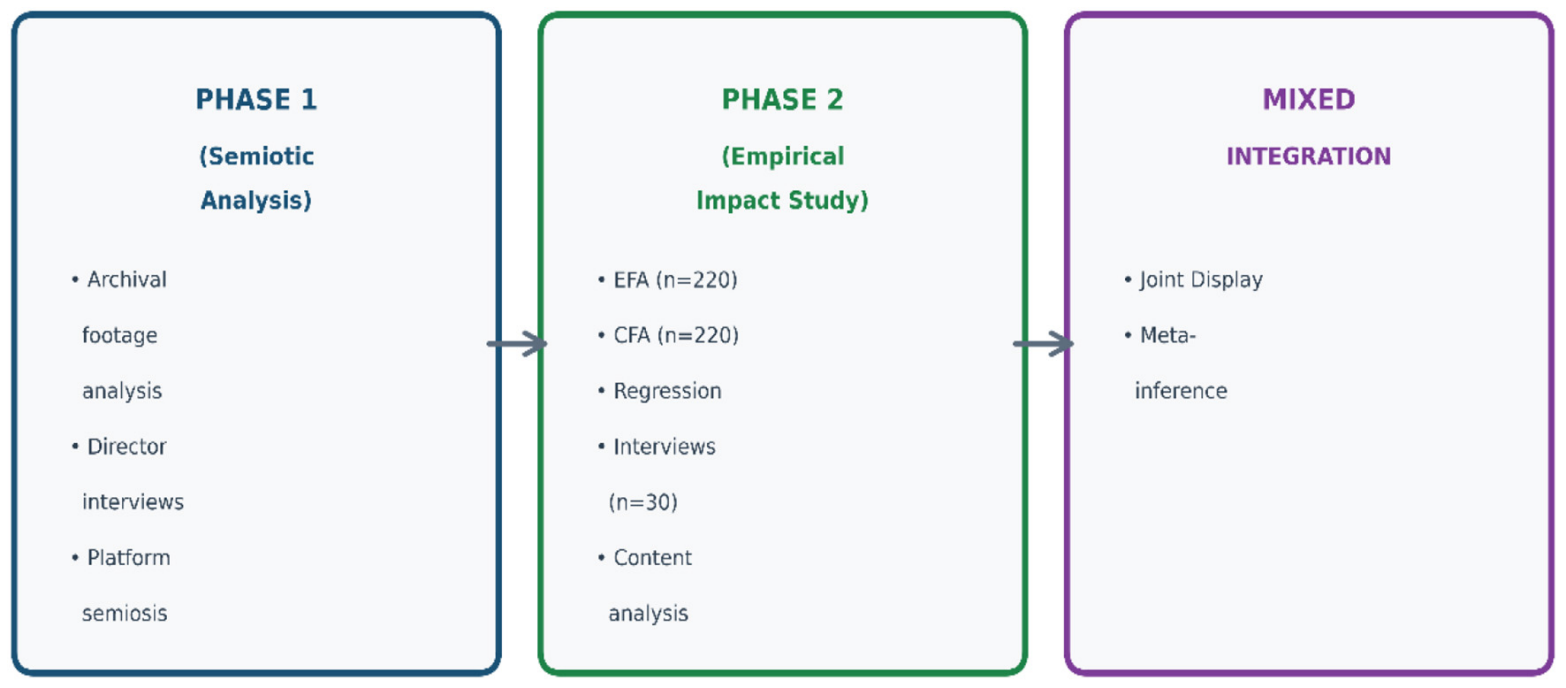
Research design.

This study uses an explanatory sequential mixed method (Creswell and Plano Clark, 2017). In this design (Figure 1), quantitative data is first collected and analyzed; qualitative data is then used to explain and deepen the quantitative findings. Integration is carried out with joint display analysis.

#### 3.2.1 Quantitative stage: sample and scale development

##### 3.2.1.1 Sampling and data collection

The universe consists of all students and academic staff at Near East University (NEU) in the 2023–2024 academic year who are watching the documentary *Yollara Düştük*. A total of 440 participants were selected by stratified random sampling (Table 1). The sample was randomly divided into two groups as per the scale development procedure:

- EFA sample: n1 = 220
- CFA sample: n_2_ = 220

**Table 1:** Quantitative sample demographics.

Variable	Category	EFA (*n* = 220)	CFA (*n* = 220)	Total (*N* = 440)
Status	Student	180 (81.8%)	178 (80.9%)	358 (81.4%)
	Instructor	40 (18.2%)	42 (19.1%)	82 (18.6%)
Gender	Women	132 (60%)	128 (58.2%)	260 (59.1%)
	Male	88 (40%)	92 (41.8%)	180 (40.9%)
Age	18–21	95 (43.2%)	92 (41.8%)	187 (42.5%)
	22–25	72 (32.7%)	75 (34.1%)	147 (33.4%)
	26+	53 (24.1%)	53 (24.1%)	106 (24.1%)
Department	Faculty of Education	48 (21.8%)	46 (20.9%)	94 (21.4%)
	Faculty of Communication	42 (19.1%)	44 (20%)	86 (19.5%)
	Social Sciences	38 (17.3%)	36 (16.4%)	74 (16.8%)
	Other	92 (41.8%)	94 (42.7%)	186 (42.3%)

##### 3.2.1.2 Scale development process

*Creating an Item Pool (45 items):*

- Literature review (Özkan and Arikan, 2009; Küçükosmanoglu, 2015; Gramsci, 1971; Hall, 1980; Nichols and Baron, 2024; Pellitteri, 2024).
- Seven expert opinions (two cinema, two education, two psychology, one communication).
- Five documentary filmmaker consultations.

*Pilot:* pre-application on 50 participants; intelligibility and face validity check.

*Item Elimination Criteria:*

- Adjusted item-total correlation: < 0.30.
- Multicollinearity: VIF > 5.
- Factor load in EFA: < 0.50 or cross load >0.40.

#### 3.2.2 Scale items and factor loads

Table 2 presents the items and factor loadings of the Documentary Cinema Educational Impact Scale (BSES).

**Table 2:** Documentary cinema Educational Impact Scale (BSES) items and factor loads (EFA, *n* = 220, Varimax rotation).

Dimension	Item	Factor load	*h* ^2^	CITC
Factor 1: perception of censorship and attitude against it (10 items, α = 0.89)				
	S1. After watching this film, I understand better how censorship restricts freedom of artistic expression	0.82	0.71	0.76
	S2. The reuse of archival footage allows historical events to be seen from different angles	0.79	0.68	0.73
	S3. Censorship practices lead to the erasure of social memory	0.85	0.75	0.79
	S4. Documentary cinema is an effective tool in questioning hegemonic narratives	0.78	0.66	0.72
	S5. The film “Yollara Düştük” reproduced the meaning of the events of 1977	0.81	0.70	0.75
	S6. Digital platforms play a significant role in disseminating censored content	0.74	0.61	0.69
	S7. The struggle of cinema workers is a part of the struggle for individual freedoms	0.77	0.66	0.71
	S8. Watching anti-censorship films increases social awareness	0.80	0.72	0.75
	S9. State intervention in artistic production weakens democratic participation	0.76	0.63	0.70
	S10. To understand historical events, it is necessary to consult various sources (archives, testimony)	0.73	0.60	0.68
Factor 2: educational motivation (eight items, α = 0.87)				
	M1. This film inspired me to tackle the challenges I faced in my education	0.83	0.74	0.77
	M2. The determination of the participants of the 1977 march was a source of motivation for me to achieve my goals	0.80	0.71	0.74
	M3. The film reminded me of the importance of solidarity and cooperation in the education process	0.78	0.67	0.72
	M4. Watching documentaries helped me approach my classes from a different, more critical perspective	0.81	0.72	0.75
	M5. The struggles of cinema workers strengthened my determination to work toward academic success	0.75	0.63	0.69
	M6. The film reminded me of my responsibility to raise individuals who are sensitive to social problems	0.79	0.69	0.73
	M7. Obstacles encountered in education can be overcome through collective action, as in this movie	0.77	0.66	0.71
	M8. Documentary cinema makes my learning process more meaningful and engaging	0.82	0.73	0.76
Factor 3: beliefs of success in life (six items, α = 0.85)				
	B1. After watching this movie, I believe more that I can overcome the difficulties in my life	0.84	0.75	0.78
	B2. Like the cinema workers in 1977, I learned to say “no” in my life	0.79	0.68	0.73
	B3. The film showed that not only individual effort but also social support and solidarity are necessary for success	0.81	0.71	0.75
	B4. The failures in my educational life found meaning again through the examples of struggle in this film	0.76	0.63	0.70
	B5. To be successful in life, it is necessary to take action at the right time rather than waiting for the right time	0.80	0.70	0.74
	B6. This film gave me the strength to face the challenges I would encounter in my future career	0.83	0.74	0.77

CITC, Corrected item-total correlation; h^2^, Communality.

All factor loadings are significant at *p* < 0.001.

#### 3.2.3 Confirmatory Factor Analysis (CFA, n = 220)

**Figure 2:**
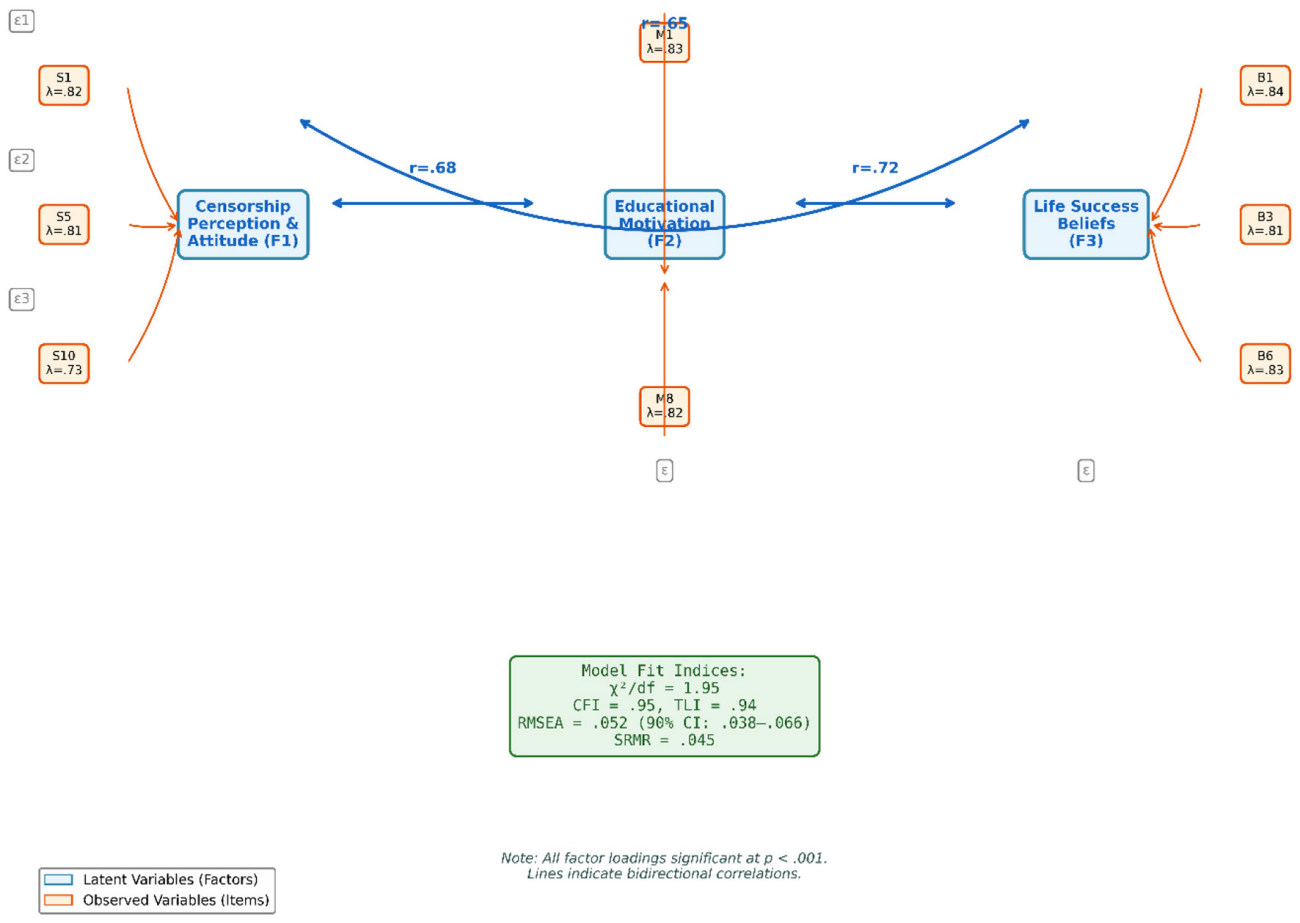
CFA path diagram (standardized coefficients).

Table 3 presents the fit indices for the Confirmatory Factor Analysis model. The CFA Path Diagram (Standardized Coefficients) is shown in Figure 2.

**Table 3:** CFA model fit indices.

Index	Value	Criteria	Assessment
χ^2^/df	1.95	< 3	Excellent
RMSEA	0.052	< 0.06	Good
CFI	0.95	>0.90	Excellent
TLI	0.94	>0.90	Excellent
SRMR	0.045	< 0.08	Good

#### 3.2.4 Validity and reliability analysis

Validity and Reliability Analysis is presented as unified and discriminant validity in Table 4.

**Table 4:** Convergent and discriminant validity.

Dimension	AVE	CR	MSV	ASV	HTMT (F1–F2)	HTMT (F1–F3)	HTMT (F2–F3)
F1: perception of censorship	0.59	0.92	0.46	0.41	—	0.71	0.69
F2: educational motivation	0.61	0.93	0.46	0.39	0.71	—	0.74
F3: beliefs of success in life	0.63	0.91	0.42	0.37	0.69	0.74	—

AVE, average variance extracted; CR, composite reliability; MSV, maximum shared variance; ASV, average shared variance; HTMT, heterotrait-monotrait ratio.

All values meet the Fornell-Larcker and HTMT criteria (AVE > CR > MSV; HTMT < 0.85).

*Reliability:*

- Cronbach's α: F1 = 0.89, F2 = 0.87, F3 = 0.85, Total = 0.91.
- Test-retest (*n* = 50, 4 weeks apart): *r* = 0.87, *p* < 0.001.
- McDonald's ω: F1 = 0.90, F2 = 0.88, F3 = 0.86.

### 3.3 Qualitative stage

#### 3.3.1 Participants and sampling

Thirty participants were selected using maximum variation sampling, and their demographic features are shown in Table 5.

**Table 5:** Qualitative sample demographics.

Variable	Category	*n*
Status	Student	24
	Instructor	6
Gender	Women	18
	Male	12
Department	Education	8
	Communication	7
	Social Sciences	6
	Other	9

#### 3.3.2 Data collection and analysis

- *Interviews:* semi-structured, 30–45 min, audio recording + note-taking
- *Content analysis:* analysis of film content in terms of three dimensions (censorship, motivation, success)
- *Coding:*
  - Two coders (Cohen's Kappa =0.84)
  - Thematic analysis (Braun and Clarke, 2006)
  - NVivo 14 software (Lumivero, 2023)

## 4 Phase 1 results: semiotic analysis

### 4.1 Close audiovisual analysis of Yollara Düştük

*Sequence A: opening sequence (00:00–03:15)*

The documentary opens with repeated images of closed cinema doors and empty exhibition spaces. The shots are predominantly static medium-long shots characterized by minimal camera movement. At the denotative level, the images simply depict inaccessible cultural venues. At the connotative level, however, they symbolize exclusion, institutional control, and restricted circulation of cultural expression.

The absence of non-diegetic music is particularly significant. Silence functions not merely as the absence of sound but as a semiotic resource that reinforces themes of suppression and invisibility. Through repetition and slow pacing, the sequence establishes an affective atmosphere of restriction before any explicit discussion of censorship occurs.

*Sequence B: Archival March Montage (12:40–18:10)*

The documentary's most significant montage sequence combines archival footage of the 1977 march with contemporary interviews. Here the archival images initially function as Peircean indexes because they retain a material connection to historical events. Through editing, however, these images become symbols of democratic resistance.

The montage repeatedly alternates between historical footage and present-day testimony. This editing strategy creates a temporal bridge that encourages viewers to interpret the march not as a completed historical event but as an ongoing political struggle.

Meaning therefore emerges through montage rather than through individual images. The sequence demonstrates how archival traces are transformed into contemporary political memory.

*Sequence C: Censorship and Festival Sequence (43:00–49:00)*

The documentary constructs censorship through an audiovisual strategy of interruption. Interview fragments are intercut with documents, newspaper headlines, and narration pauses.

Editing rhythm slows considerably compared with earlier sections. The discontinuous structure mirrors the institutional interruption of circulation caused by censorship practices.

The interaction between testimony and archival evidence creates a layered representational strategy in which personal memory and documentary proof reinforce each other. Rather than presenting censorship as an abstract concept, the sequence materializes censorship through audiovisual form.

### 4.2 Counter-hegemony and semiotic resistance in Yollara Düştük

*Yollara Düştük* embodies semiotic resistance in the face of censorship. The documentary archives the semiotic traces of censorship practices in the 1960s−70s, documents the prevention of circulation in institutional spaces (festivals), and resists bureaucratic censorship through digital platform circulation.

Circulation to digital platforms semiotically involves these transformations:

- *Spatial expansion:* transition from the physical and institutional boundaries of festivals to the “limitless” semiosphere of the internet.
- *Temporal expansion:* the “open” time structure of archival footage from the 1977 context to the 2014 production, then to the 2019 digital broadcast and ongoing audience commentary.
- *Social expansion:* transformation from passive spectators to “semiotic actors” who interpret and share.

This process is an update of Eco's (1976) concept of “open work” in the digital age: the documentary becomes a semiotic space constantly produced and negotiated, rather than a completed meaning.

### 4.3 Hypothesis testing: semiotic phase

H1 is semiotically confirmed: the recontextualization of archival images produces new layers of meaning at the level of Peirce's “symbol,” enabling the “disassembly” of hegemonic myths. The images of the 1977 march could have had different symbolic meanings in their original contexts (news bulletins and the period's propaganda). However, Yeşil's montage repositions these images within the narrative of “resistance to censorship,” creating a symbolic structure that opposes the hegemonic myth that “Turkish cinema is apolitical.”

The analytical procedure for this confirmation involved the following steps: (a) identification of archival images in their original 1977 context (newsreels, state television broadcasts) through archival research; (b) comparison of original contextual meaning with the meaning produced by montage in Sequence B; (c) coding of symbolic transformation according to the four-dimension analytical framework (visual composition, montage structure, sound design, semiotic function); (d) triangulation with secondary historical sources on the 1977 march. The archival images of the march, originally coded as “labor dispute” or “industrial action” in 1977 news discourse, were recoded through juxtaposition with 2014 testimonies as “democratic resistance against censorship” and “collective memory of Turkish cinema.”

(Denić 2024) examines the “semiotic performance of the negotiation of belonging” in first-person documentaries in Latin America, while showing that the reuse of archival materials is “a dialogue of subjective memory with collective memory.” In *Yollara Düştük*, this dialogue creates a semiotic tension between individual testimonies (2014 interviews) and collective action (1977 March).

Karimi (2024) examines how “gender dynamics are semiotically hidden and revealed” in Iranian cinema, showing how the meanings made “invisible” by censorship can be brought to light through alternative semiotic strategies. A similar strategy works in *Yollara Düştük*: the struggle of cinema workers, which has been made “invisible” by official history, is brought to light again through the semiosis of archival footage.

H2 is also semiotically confirmed: circulation on digital platforms represents an expansion from the limited production of meaning to unlimited communities of interpretation, in accordance with Eco's concept of “open work.”

The evaluation of H2 followed these criteria: (a) documentation of festival censorship preventing the documentary's institutional circulation (Antalya Golden Orange 2014, Istanbul Film Festival 2015); (b) evidence of director's deliberate choice to upload to YouTube and Vimeo as circumvention strategy; (c) analysis of user comments, shares, and secondary discourses on digital platforms as evidence of participatory meaning-making; (d) examination of view counts and geographical distribution of audience as indicators of expanded semiotic circulation. The documentary's presence on YouTube (uploaded in 2014, with ongoing commentary through 2024) satisfies these criteria, demonstrating a shift from a closed festival semiosphere to an open digital semiosphere.

However, this expansion does not operate semiotically within “absolute freedom” but within “new regulations.” Poell et al. (2024) show that in the age of the platform, “the semiotic regimes of cultural production become complex”: platforms simultaneously function as mechanisms that (a) enable the circulation of counter-hegemonic meanings and (b) control the algorithmic visibility of these meanings.

Cardullo and Kitchin (2025) examine how platform governance shapes practices of “semiotic citizenship” and how participatory meaning production is constrained by “platform logic.” The presence of *Yollara Düştük* on YouTube embodies this dual dynamic—while the video is accessible, algorithmic suggestions, comment moderation, and copyright checks semiotically create “visibility hierarchies.”

Turten (2022) highlights the “semiotic circulation of documentaries censored in Turkey on a global scale through digital platforms.” However, Akser and Baybars (2022) remind us that these platforms “are not completely free spaces, but carry new risks of censorship and control.”

H3 posits that the director's practice is a semiotic intervention within Hall's “oppositional coding” model. Yeşil's practice includes three semiotic interventions:

(a) *Archival selection:* semiotic preference for which images to include as “traces of reality.” The choice of “well-known faces” such as Tarik Akan and Fatma Girik enhances the narrative's iconic and symbolic impact.
(b) *Montage strategy:* generating new semantic relationships through syntagmatic and paradigmatic images. The combination of 1977 images and 2014 testimonies creates an “intertemporal” semiotic structure.
(c) *Circulation strategy:* the rejection of the festival space and the choice of digital platforms is a semiotic preference for the “open” circulation of meaning.

The confirmation of H3 was operationalized through the following analytical steps: (a) identification of director's editing decisions in the three selected sequences; (b) comparison of Yeşil's montage choices with conventional hegemonic representations of Turkish cinema history in state television archives; (c) analysis of circulation strategy as deliberate counter-hegemonic intervention (rejection of Operational Certificate, upload to digital platforms); (d) coding of these practices within Hall's (1980) encoding/decoding framework as “oppositional coding” rather than “preferred” or “negotiated” coding. The director's selection of archival footage excluded state-sponsored representations of the 1977 march as “disorder” or “illegal gathering” and instead recoded these images through juxtaposition with celebrity testimonies as “legitimate democratic resistance.”

This practice parallels the findings of Scotini et al. (2017) in their “documentary and archival” studies: documentary cinema, as the “semiotic stage of the politics of memory,” challenges hegemonic narratives by reinterpreting archival materials.

The example of Turkey shows that censorship operates semiotically in a “continuity and transformation” mode. Compared with the film regulations in France (CNC, 2024), practices in Turkey exhibit a stronger semiotic convergence between “national identity discourse” and “exhibition control.”

In examining the “semiotic differences of visual media policy” in Europe, Scotini et al. (2017) show that regulatory frameworks are determined not only by their level of restrictiveness, but also by semiotic differences in “normative justification structures.” In Turkey, ambiguous categories such as “national interest” and “public morality” allow the semiotic reproduction of hegemonic “common sense.”

The Black Tape initiative is a semiotic documentation and questioning of this process. Black Tape (2024) documents the semiotic tension between “freedom of expression” and “national security,” making visible the strategies censorship uses to gain “legitimacy.” Pellitteri (2024) shows that such initiatives create “counter-memory infrastructures” in opposition to “hegemonic memory infrastructures.”

## 5 Phase 2 results: empirical educational impact

### 5.1 Quantitative findings

#### 5.1.1 Descriptive statistics and normality

Descriptive Statistics for Scale Scores and Normality Tests are shown in Table 6.

**Table 6:** Descriptive statistics of scale scores and normality tests.

Dimension	*N*	$\bar{x}$	SD	Skewness	Kurtosis	K-S	S-W
F1: perception of censorship	440	3.72	0.74	−0.28	−0.35	0.04	0.98
F2: educational motivation	440	3.95	0.68	−0.42	−0.22	0.05	0.97
F3: beliefs of success in life	440	4.08	0.62	0.38	−0.18	0.06	0.96
Total	440	3.88	0.58	−0.22	−0.25	0.04	0.98

K-S, Kolmogorov–Smirnov; S-W, Shapiro–Wilk.

All tests are *p* > 0.05, assuming normal distribution.

#### 5.1.2 Demographic differences

Table 7 presents the Comparison of Scale Scores according to demographic variables.

**Table 7:** Comparison of scale scores according to demographic variables.

Variable	Group	*N*	$\bar{x}$	SD	*t/f*	*p-*value	Cohen's *d*/η^2^	95% CI
Status	Student	358	3.85	0.59	*t*_(438)_ = −2.89	0.004	0.42	−0.38, −0.08
	Instructor	82	4.02	0.52				
Gender	Women	260	3.92	0.56	*t*_(438)_ = −3.12	0.002	0.38	−0.42, −0.10
	Male	180	3.82	0.61				
Age	18–21	187	3.78	0.65	*F*_(2, 437)_ = 4.85	0.008	0.022	—
	22–25	147	3.89	0.58				
	26+	106	4.01	0.52				
Department	Faculty of Education	94	4.05^a^	0.51	*F*_(3, 436)_ = 5.42	0.001	0.036	—
	Faculty of Communication	86	3.98^ab^	0.54				
	Social Sciences	74	3.85^b^	0.62				
	Other	186	3.76^b^	0.63				

Superscript indicates that the difference between different letters is significant at the *p* < 0.05 level (Bonferroni correction).

#### 5.1.3 Multiple regression analysis

The results of the Multiple Regression Analysis are shown in Table 8.

**Table 8:** Variables predicting scale scores (multiple regression).

Variable	*B*	SE	β	*t*	*p-*value	VIF
(Constant)	2.65	0.22	—	12.05	< 0.001	—
Status (Instructor = 1)	0.32	0.08	0.24	4.00	< 0.001	1.12
Gender (Female = 1)	0.18	0.06	0.15	3.00	0.003	1.08
Age	0.12	0.04	0.14	3.00	0.003	1.05
Department (Education = 1)	0.22	0.09	0.12	2.44	0.015	1.15
Had heard of it before (Yes = 1)	0.08	0.07	0.05	1.14	0.255	1.03

R^2^ =0.28, F_(5, 434)_ = 33.85, *p* < 0.001, Adjusted R^2^ = 0.27, Durbin–Watson = 2.01. VIF values < 5, no multicollinearity problem.

### 5.2 Qualitative findings

#### 5.2.1 Thematic analysis (Interviews)

*Theme 1: “Ignored History”*

“I had never heard of the 1977 march before watching the movie. These subjects are not taught at school. The film showed how history is written and how things are hidden.” *(K3, Student, Faculty of Education)*

*Theme 2: “Solidarity and Motivation”*

“The gathering of cinema workers in 1977 gave me the message ‘you are not alone'. I remember this movie when I had difficulty in my educational life.” *(K12, Student, Communication)*

*Theme 3: “Actuality of Censorship”*

“The film is about 1977, but it is as if it is about today. Censorship still exists, only its form has changed. Digital platforms have opened up a new space, but there is no complete freedom.” *(K18, Instructor, Communication)*

*Theme 4: “Fighting for Success”*

“Tarik Akan's determination… In order to be successful in education, it is not enough to just study; it is necessary to take a stance and struggle.” *(K7, Student, Social Sciences)*

#### 5.2.2 Student opinions about the documentary

Beyond the structured interview themes, students expressed diverse opinions about the documentary's impact on their personal and academic lives:

*On Historical Awareness:*

“Before this film, I thought Turkish cinema was just about entertainment. Seeing Tarik Akan, Cüneyt Arkin, and Fatma Girik marching for freedom changed my entire perspective. They weren't just actors — they were citizens fighting for their rights.” *(K5, Student, Faculty of Education)*

*On Emotional Impact:*

“I cried during the testimony scenes. When Türkan Soray said ‘it had to end and explode somewhere,' I felt that explosion in myself. It made me question what I would do if I faced similar censorship in my own field.” *(K14, Student, Communication)*

*On Educational Motivation:*

“When I saw that 400 people walked for three days from Istanbul to Ankara, my own academic challenges seemed smaller. If they could do that, I can finish my thesis.” *(K9, Student, Social Sciences)*

*On Digital Resistance:*

“The most powerful part for me was learning that the director uploaded the film to YouTube because the festivals wouldn't show it. That's real resistance—using technology to fight censorship. It made me think about how I can use digital platforms for my own activism.” *(K21, Student, Communication)*

*On Professional Identity (Education Students):*

“As a future teacher, this film showed me that education is not neutral. What we teach and what we hide from students are forms of censorship. I want to be a teacher who shows students the hidden histories.” *(K2, Student, Faculty of Education)*

*On Gender Perspectives:*

“I noticed that women like Türkan Soray and Fatma Girik were at the front of the march. In a male-dominated industry, their presence was powerful. It motivated me as a woman in academia to stand up for my beliefs.” *(K16, Student, Social Sciences)*

*On Cynicism vs. Hope:*

“Part of me thinks nothing has changed—we still have censorship today. But another part feels hopeful because at least now we have films like this that document the struggle. The film gave me both anger and hope.” *(K25, Student, Other)*

### 5.3 Mixed integration: joint display analysis

Joint display: integration of quantitative and qualitative findings is shown in Table 9.

**Table 9:** Joint display: integration of quantitative-qualitative findings.

Quantitative finding (*N* = 440)	Qualitative finding (*n* = 30)	Meta-inference
Educational motivation was the highest mean ($\bar{x}$ = 3.95)	“Film has been my inspiration when I have struggled” (K12)	Documentary cinema strongly increases motivation at the individual level
Instructors have higher scores (d = 0.42)	“Censorship still exists, only its form has changed” (K18)	The difference in status reflects structural awareness; Lecturers combine both motivation and structural criticism
Women had higher scores (*d* = 0.38)	Female participants used stronger expressions in empathy and emotional connection	The gender difference can be explained by the level of emotional functioning and empathy
Faculty of Education highest ($\bar{x}$ = 4.05)	Education students emphasized the responsibility of “being a teacher for society”	Professional identity reinforces the educational message of the film
Points increase with age (η^2^ = 0.022)	Older participants stated that they had a better grasp of the historical context	Age is associated with historical awareness and life experience

## 6 Discussion

### 6.1 Integration of semiotic and empirical findings

This study demonstrates that *Yollara Düştük* operates simultaneously as a semiotic intervention and an educational catalyst. The semiotic analysis (Phase 1) reveals how the documentary dismantles hegemonic myths through the recontextualization of archival footage, while the empirical analysis (Phase 2) quantifies the documentary's impact on viewers' motivation, perceptions of censorship, and success beliefs.

The most critical finding was that **e**ducational motivation had the highest mean ($\bar{x}$ = 3.95). This suggests that documentary cinema is a strong source of motivation at the individual level (Küçükosmanoglu, 2015; Wildwood Media, 2025). The documentary's ability to transform historical struggle into personal inspiration aligns with Bandura's (1977) self-efficacy theory: observing the success stories of others (vicarious experience) can increase one's beliefs in one's own success. The high average of the “beliefs in success in life” dimension ($\bar{x}$ = 4.08) shows that the film fulfills this function.

The higher scores of the instructors (*d* = 0.42) can be explained by structural awareness and critical perspective. Lecturers can analyze the film not only as an emotional experience, but also within the framework of hegemony and censorship theories. This situation parallels Gramsci's (1971) concept of “organic intellectual.”

The higher score of female participants (*d* = 0.38) can be explained by the tendency to emotional functioning and empathy in documentary cinema. Previous studies indicate that female audiences experience higher emotional engagement in documentaries on social issues (Barchas-Lichtenstein et al., 2020). In particular, significant increases were observed in gender bias awareness (*r* = 0.31), empathy (*r* = 0.26), and perspective-taking (*r* = 0.35) among female participants who watched the documentary “Picture a Scientist” (Barchas-Lichtenstein et al., 2020).

### 6.2 Yollara Düştük and counter-hegemonic meaning production

The film creates a counter-hegemonic meaning space by retelling the 1977 march through archival footage and current testimonies. Within the framework of Barthes's (1972) concept of “myth,” the film questions the “apolitical” myth of Turkish cinema. The recontextualization of archival images embodies Peirce, 1931–1958 process of symbolic recoding of indexical traces (Kruk, 2025).

The semiotic analysis demonstrates this process through concrete audiovisual evidence. In Sequence B, the archival footage of the 1977 march—originally functioning as indexical traces of a specific labor action—undergoes symbolic recoding through montage with 2014 testimonies. The editing rhythm, alternating between collective march footage and individual testimony shots, constructs a dialectical relationship between past and present, transforming the historical event into an ongoing symbol of resistance. This operationalizes the theoretical claim that archival footage is “extracted from its original indexed context and subjected to symbolic re-encoding”.

Joint display analysis shows that the film is effective at conveying an understanding of historical events, but it falls short. This coincides with the findings of Özkan and Arikan (2009): University students in Turkey exhibit contradictory attitudes toward censorship; despite high approval rates, it is undecided at the level of implementation (Özkan and Arikan, 2009). Research conducted by the Speak Up Platform (2018) with 350 participants in Turkey shows that censorship is a multidimensional process encompassing not only the media but also art, education, and daily life (Speak Up Platform, 2018).

### 6.3 Educational motivation and beliefs of success in life

The film strengthens the audience's belief in self-sufficiency through the struggle of the cinema workers in 1977. A UK study found that film education had the greatest impact on students' “attitude/motivation to learn”; 83% said that film use encouraged creative thinking, and 84% said film could reach students with a wider range of abilities (Teaching Times, 2023). These findings support the high average of the “educational motivation” dimension of BSES.

The student opinions reveal a nuanced picture: while students felt personally motivated by the documentary, many remained skeptical about whether individual motivation could translate into structural change. This tension between personal inspiration and structural cynicism mirrors the documentary's own position—it can inspire individuals but cannot dismantle hegemonic censorship structures on its own.

### 6.4 The ironic censorship of a documentary about censorship

Perhaps the most striking aspect of this study is the meta-narrative of censorship. The documentary *Yollara Düştük*, which documents the historical censorship of Turkish cinema, itself became a victim of contemporary censorship mechanisms. The refusal of the Ministry of Culture and Tourism to grant an “Operational Certificate” without clear criteria, the festival system's reluctance to screen politically sensitive documentaries, and the eventual necessity of uploading the film to YouTube—all of these constitute a living example of the very phenomenon the documentary seeks to document.

This recursive structure creates what we might call a “semiotic mirror”: the documentary reflects censorship while simultaneously being reflected by it. For students and faculty watching the film, this mirror effect was palpable. As one student noted:

“The film is about censorship, and then I learned it was censored itself. That's when I really understood what censorship means—it's not just history, it's happening right now, to this very film.” *(K11, Student, Faculty of Communication)*

This recursive censorship narrative proved to be one of the most powerful educational tools in the documentary. It transformed abstract theoretical concepts about hegemony and censorship into concrete, observable reality.

### 6.5 Digital platforms as educational and semiotic spaces

The circulation of *Yollara Düştük* on digital platforms represents what Eco would term an “open work”—a text that is constantly reinterpreted and renegotiated by its audience. For university students, this digital accessibility was crucial. Many participants first encountered the documentary through YouTube, not through formal educational channels.

However, as the semiotic analysis reveals, digital platforms are not neutral spaces. Algorithmic filtering, content moderation, and copyright enforcement create new forms of “soft censorship” that regulate which meanings become visible. Students recognized this duality:

“YouTube let us see the film, but who decides what else we don't see? The algorithm is the new censor.” *(K19, Student, Social Sciences)*

This awareness—that digital liberation carries its own constraints—represents a sophisticated understanding of contemporary media ecology that the documentary helped cultivate.

### 6.6 . Structural limits of documentary cinema as counter-hegemonic practice

#### 6.6.1 The tension between symbolic awareness and structural transformation

The central question raised by this study is whether documentary cinema can function as more than a symbolic intervention—whether it can translate semiotic disruption into structural or institutional change. Our findings suggest a complex and ultimately limited answer. While *Yollara Düştük* successfully dismantles hegemonic myths at the symbolic level (H1 confirmed), produces new interpretive communities through digital circulation (H2 confirmed), and intervenes in hegemonic discourse through directorial practice (H3 confirmed), these achievements remain confined to the realm of meaning-production rather than material power.

The quantitative data reveal that the documentary's strongest effect is on individual educational motivation ($\bar{x}$ = 3.95) and beliefs in personal success ($\bar{x}$ = 4.08), while its weakest effect is on the perception of structural censorship ($\bar{x}$ = 3.72). This asymmetry is theoretically significant: it indicates that the film transforms individual subjectivity more effectively than it transforms structural understanding. Participants feel more capable of overcoming personal obstacles (B1, B3, and B6) and more motivated in their educational pursuits (M1, M2, and M4), yet they do not significantly alter their understanding of censorship as an institutional system (S1, S3, and S9).

This finding aligns with what we term the “*motivation-action gap”* in documentary reception: the film generates affective and cognitive engagement without necessarily producing the political behaviors—collective mobilization, institutional advocacy, policy intervention—that would constitute structural transformation. As one participant candidly observed:

“*Part of me thinks nothing has changed.” (K25)*

This ambivalence is not a failure of the documentary but rather a structural condition of documentary cinema under late capitalism, in which symbolic production is increasingly decoupled from the political economy.

#### 6.6.2 Comparative perspectives: is the counter-hegemonic impact of Yollara Düştük generalizable?

To assess whether the effects observed in this study are specific to *Yollara Düştük* or generalizable to other censored documentaries, we briefly situate our findings within the broader landscape of politically engaged documentary cinema.

The Iranian documentary *The Circle* (Panahi, 2000), banned in its home country but circulated internationally, similarly employed indexical footage of Tehran's streets to symbolically challenge gendered censorship; yet its impact remained largely confined to festival audiences and diasporic communities, with limited domestic structural effect (Karimi, 2024). In contrast, the Chilean documentary *The Battle of Chile* (Patricio Guzmán, 1975–79), produced under conditions of explicit state censorship, became a direct organizational tool for anti-Pinochet solidarity networks, demonstrating that under specific historical conjunctures—when documentary circulation is tied to organized social movements—symbolic awareness can indeed translate into structural action.

The Turkish case occupies an intermediate position. *Yollara Düştük* was produced in a context of “*soft authoritarianism”* (Akser and Baybars, 2022), in which formal democratic institutions persist but are progressively hollowed out. Unlike Pinochet's Chile, where censorship was absolute and therefore produced unified resistance, or contemporary Iran, where censorship is religiously legitimized and therefore produces diasporic counter-publics, Turkey's ambiguous regulatory environment produces what we might call “*semiotic fatigue”*: audiences recognize censorship, are moved by its documentation, yet remain uncertain about actionable responses. This comparative perspective suggests that the counter-hegemonic impact of compilation documentaries is not universal but is mediated by the specific “*censorship regime”* (narrow vs. broad, explicit vs. diffuse) within which they circulate.

#### 6.6.3 Emotional engagement vs. structural transformation: the role of critical media literacy

The question of whether audience motivation inevitably results in sustained critical action or political engagement is central to a core debate in media effects research. Our qualitative data reveal a tripartite pattern of audience response that complicates simple linear models of documentary impact:

(a) *Emotional identification:* participants report strong empathetic connections with the 1977 marchers (“*I cried during the testimony scenes*,” K14). This affective engagement is immediate and visceral, operating at what Tompkins (1995) calls the “*affective-cognitive interface*.”
(b) *Cognitive reframing:* participants demonstrate new critical perspectives on Turkish cinema history (“*Before this film, I thought Turkish cinema was just about entertainment*,” K5). This cognitive transformation involves the “disassembly” of hegemonic myths, as documented by the semiotic analysis.
(c) *Behavioral ambivalence:* despite (a) and (b), participants express uncertainty about translating awareness into action. The education student who vows to “show students the hidden histories” (K2) represents the minority; more typical is the participant who feels “both anger and hope” yet acknowledges that “nothing has changed” (K25).

This pattern suggests that emotional engagement and cognitive transformation are necessary but insufficient conditions for structural political action. What is missing, our data imply, is what critical media literacy scholars' term “*action competence”* (Jenkins et al., 2016): the pedagogical scaffolding that helps viewers move from “*reading”* documentary texts critically to “*acting”* upon their readings politically. Without such scaffolding—structured discussion, collective analysis, organizational affiliation—documentary cinema risks functioning as what Adorno (1951) critically termed “*culture industry”:* a form of symbolic politics that absorbs dissent without transforming structures.

Our findings thus underscore the importance of integrating documentary screening with critical media literacy pedagogy. The BSES instrument, particularly its “*Educational Motivation”* dimension, could be expanded in future research to include items measuring action competence and political self-efficacy, thereby bridging the gap between individual motivation and collective transformation.

#### 6.6.4 Digital platforms: democratization or relocated censorship?

The question of whether digital platforms genuinely democratize marginalized voices or simply relocate censorship into more subtle algorithmic forms requires sustained theoretical engagement. Our data support a dialectical conclusion.

On the one hand, digital circulation enabled *Yollara Düştük* to bypass the “*Operational Certificate” r*equirement and reach audiences that festival censorship would have excluded. The platform logic of YouTube—low barrier to entry, global accessibility, user-generated commentary—does represent a genuine semiotic expansion compared with the gatekept spaces of state-regulated festivals. As Eco's concept of “*open work”* predicts, the documentary becomes a site of ongoing interpretive negotiation rather than a closed authorial product.

On the other hand, our qualitative data and theoretical analysis converge on the recognition that platform governance introduces new forms of “*soft censorship”* that, in some respects, are more insidious than explicit state prohibition. Algorithmic filtering, as Cardullo and Kitchin (2025) demonstrate, does not ban content but makes it statistically invisible; copyright enforcement does not censor speech but monetizes and thereby controls its circulation; content moderation does not prohibit dissent but subjects it to corporate terms of service. The student's observation that “*the algorithm is the new censor”* (K19) is not merely metaphorical but describes a real transformation in the modalities of semiotic control.

This dialectic has important implications for documentary practice. Director Yeşil's choice to upload to YouTube was tactically necessary but strategically incomplete. Future documentary activism must attend not only to content production but also to platform governance: advocating for transparent algorithmic auditing, supporting decentralized hosting alternatives (e.g., PeerTube, Internet Archive), and building “*counter-memory* infrastructures” (Pellitteri, 2024) that are less vulnerable to platform capitalism's visibility hierarchies.

#### 6.6.5 Audience reception and ideological negotiation: toward a critical media literacy framework

Finally, broader perspectives need to be addressed from critical media literacy and audience perception studies. Hall's (1980) coding/decoding model provides the basic framework we used in Phase One, but we are now expanding it through interaction with more recent research.

Morley (1980) ethnographic audience studies demonstrated that decoding positions are not fixed by textual structure but are negotiated through viewers' social locations, cultural competencies, and interpretive communities. Our data confirm this: instructors decoded the film through “*structural”* lenses (hegemony, censorship regimes), while students decoded it through “*experiential”* lenses (personal motivation, educational obstacles). These differential decodings have political consequences: the instructor who recognize*s “censorship still exists, only its form has changed”* (K18) is more likely to engage in structural critique than the student who draws personal inspiration from Tarik Akan's determination (K7).

Livingstone (2004) work on media literacy and democratic citizenship further clarifies this dynamic. She argues that critical media consumption requires not only “*access”* and “*understanding”* but also “*participation”* and “*creation.” O*ur BSES instrument primarily measures the first two dimensions; future research should incorporate measures of the latter two to assess whether documentary viewers move from passive reception to active production of counter-hegemonic content.

Couldry and Hepp (2017) concept of “*deep mediatization”* offers a final theoretical refinement. They argue that in fully mediatized societies, the distinction between “*media”* and “*reality”* collapses: social institutions are increasingly constituted through media practices. In this context, documentary cinema is not merely a representation of censorship but itself a social institution that enacts relations of censorship. The recursive structure of *Yollara Düştük*—a film about censorship that is itself censored—exemplifies this deep mediatization: the documentary does not merely document power relations but performs them, making visible the constitutive role of media in the production of political reality.

### 6.7 Limitations

- *Sample limitation:* only one university (EMU) and a single film.
- *Self-report bias:* the effect of social desirability.
- *Temporal limitation:* inability to establish causality due to cross-sectional design.
- *Language limitation:* the Turkish scale should be adapted to other languages.
- *Semiotic limitation:* phase 1 relies on interpretive analysis that may reflect the researcher's subjectivity.
- *Structural limitation:* the study design cannot capture whether individual-level motivational changes translate into collective political action or institutional reform.

### 6.8 Future research

- Longitudinal designs and the change of film effect over time.
- Differences in influence between different types of documentaries (compilation, direct cinema, ethnographic).
- Comparative cultural analyses (TRNC-Turkey-UK).
- Experimental designs with control groups to establish causality.
- Investigation of algorithmic censorship on digital platforms and its educational implications.
- Comparative analyses with other censored documentaries (e.g., Iranian, Chilean, Chinese cases) to assess whether counter-hegemonic impact is sustained across different censorship regimes.
- Development of action-competence measures to bridge the gap between individual motivation and collective political engagement .

## 7 Conclusion and recommendations

This two-phase study reveals the multifaceted impact of the documentary *Yollara Düştük*. *Phase 1* demonstrates that compilation documentaries serve as the semiotic stage of ideological struggle, dissolving hegemonic myths and forming new systems of political meaning. The recontextualization of archival images produces secondary semiosis within the framework of Peirce's tripartite model. Images, “traces of reality” at the indexical level, acquire new layers of meaning at the symbolic level through montage and narrative context. This process enables the semiotic “disassembly” of hegemonic myths (e.g., “Turkish cinema is apolitical”).

*Phase 2* reveals the documentary's positive impact on educational motivation and beliefs about success in life; however, it is insufficient to change perceptions of structural censorship. The Documentary Cinema Educational Impact Scale (BSES) is a valid and reliable tool, the first Turkish measurement tool in this field.

Circulation on digital platforms represents a semiotic expansion from the limited production of meaning (the closed mechanisms of festivals) to boundless communities of interpretation, in line with Eco's concept of “open work.” But this expansion is subject to new semiotic arrangements by the “visibility politics” of platform capitalism.

The director's practice functions as a semiotic intervention against hegemonic discourse within Hall's “counter-coding” model. Archive selection, montage strategy, and circulation preferences involve conscious interventions in the ideological dimension of meaning production.

Semiologically, censorship operates as a regime of “regulation of meaning”; however, this regime can be overcome by counter-hegemonic semiosis practices. *Yollara Düştük* epitomizes this practice of transcendence: by documenting censorship, resisting censorship, and creating an “open” circulation of meaning on digital platforms.

As a result, documentary cinema functions as the semiotic stage of the struggle for hegemony. Images are not only the “trace of reality,” but also the “production area of meaning.” Censorship seeks to limit this area; however, the compilation documentary, through the resemiosis of archival footage, enables the circulation of alternative systems of meaning.

From a Marxist-cultural perspective, this process operates not at the level of “*consciousness*,” but at the level of “*meaning production practices*.” Semiotic struggle does not automatically destroy hegemony; however, it questions the naturalized structure of “*common sense*,” opens up alternative reading positions, and creates spaces for collective negotiation of meaning.

Nevertheless, the semiotic achievements must be evaluated with critical modesty. Documentary cinema can disrupt hegemonic myths, cultivate critical awareness, and inspire individual motivation, but it cannot substitute for organized political action, institutional reform, or structural transformation. The “motivation-action gap” identified in our data is not a methodological artifact but a structural feature of documentary's position within contemporary media capitalism. Future research and practice must therefore attend not only to the semiotic quality of documentary texts but also to the pedagogical and organizational infrastructures that can translate symbolic awareness into political efficacy.

The digital presence of *Yollara Düştük* is indicative of this ongoing struggle: ideas, images, and meanings continue to circulate despite control mechanisms. Semiologically, “*closed*” meanings are “*opened*” in other semiospheres; suppressed discourses are reproduced with new codings.

## Data Availability

The raw data supporting the conclusions of this article will be made available by the authors, without undue reservation.
